# Cooling‐promoted myogenic differentiation of murine bone marrow mesenchymal stem cells through TRPM8 activation in vitro

**DOI:** 10.14814/phy2.15855

**Published:** 2023-12-12

**Authors:** Ryo Takagi, Junya Takegaki, Shion Osana, Yutaka Kano, Satoshi Konishi, Satoshi Fujita

**Affiliations:** ^1^ Ritsumeikan Global Innovation Research Organization Ritsumeikan University Shiga Japan; ^2^ Research Organization of Science and Technology Ritsumeikan University Shiga Japan; ^3^ Graduate School of Informatics and Engineering University of Electro‐Communications Tokyo Japan; ^4^ Faculty of Physical Education, Department of Sport and Medical Science Kokushikan University Tokyo Japan; ^5^ Center for Neuroscience and Biomedical Engineering University of Electro‐Communications Tokyo Japan; ^6^ Faculty of Science and Engineering Ritsumeikan University Shiga Japan; ^7^ Faculty of Sport and Health Science Ritsumeikan University Shiga Japan

**Keywords:** cooling, differentiation, mesenchymal stem cells, TRPM8

## Abstract

TRPM8 agonist has been reported to promote osteogenic differentiation of mesenchymal stem cells (MSCs), therefore we evaluated whether cooling‐induced activation of TRPM8 promotes myogenic differentiation of MSCs. We used 5‐azacytidine as a myogenic differentiation inducer in murine bone marrow‐derived MSCs. Addition of menthol, a TRPM8 agonist, to the differentiation induction medium significantly, increased the percentage of MyoD‐positive cells, a specific marker of myogenic differentiation. We performed intracellular Ca^2+^ imaging experiments using fura‐2 to confirm TRPM8 activation by cooling stimulation. The results confirmed that intracellular Ca^2+^ concentration ([Ca^2+^]i) increases due to TRPM8 activation, and TRPM8 antagonist inhibits increase in [Ca^2+^]i at medium temperatures below 19°C. We also examined the effect of cooling exposure time on myogenic differentiation of MSCs using an external cooling stimulus set at 17°C. The results showed that 60 min of cooling had an acceleratory effect on differentiation (2.18 ± 0.27 times). We observed that the TRPM8 antagonist counteracted the differentiation‐promoting effect of the cooling. These results suggest that TRPM8 might modulate the multiple differentiation pathways of MSCs, and that cooling is an effective way of activating TRPM8, which regulates MSCs differentiation in vitro.

## 1 INTRODUCTION

Mesenchymal stem cells (MSCs) are somatic stem cells, found in a variety of tissues, including bone marrow and adipose tissue. MSCs are multipotent and can undergo osteogenic, chondrogenic, and adipogenic differentiation, which are mesoderm‐derived cells, as well as neural and epidermal differentiation (Cho et al., 2005; Ma et al., 2009), which are ectoderm‐derived cells. They can also differentiate into hepatocyte and islet cells (Snykers et al., 2007; Zanini et al., 2011), which are endoderm‐derived cells. MSCs also have immunomodulatory properties (Kim & Cho, 2022; Uccelli et al., 2008) and have been shown to be effective in transplantation therapy for various diseases such as osteoarthritis and inflammatory diseases (Niebergall‐Roth et al., 2022; Wei & Bao, 2022). Among its cell transplantation effects, secretion of bioactive factors is the main one (Caplan & Dennis, 2006; Nakamura et al., 2015), and multipotency is maintained (Liechty et al., 2000) but scarce in vivo (Natsu et al., 2004; Phinney & Prockop, 2007). Grabowska et al. reported that after transplantation of mesenchymal cells into injured skeletal muscles, the cells fuse in only about 5% of muscle fibers (Grabowska et al., 2012). Hence, the use of stem cells can be further expanded in regenerative medicine if the differentiation potential of MSCs can be demonstrated in vivo.

MSCs possess temperature‐sensitive transient receptor potential (TRP) channels. Differentiation of MSCs is regulated by thermal stimulation and activation of the TRP channels. The effects of hyperthermia have been shown to promote myogenic (Miksiunas et al., 2022), chondrogenic (Chen et al., 2014), and osteogenic differentiation (Chen et al., 2013; Norgaard et al., 2006) in MSCs. It has also been reported that hyperthermia contributes to the maintenance of differentiation potential (Choudhery et al., 2015). TRPV2 of MSCs gets activated at temperatures above 52°C (Caterina et al., 1999), and it has been reported to affect MSCs adipogenic differentiation (Kim et al., 2021). In the low‐temperature regions, TRPM8, which is activated at mildly low temperatures (McKemy et al., 2002; Peier et al., 2002), has been reported to regulate osteogenic differentiation of MSCs (Acharya, Kumar, et al., 2021; Henao et al., 2021), but there are no reports that exposure to cooling has a differentiation‐promoting effect.

Temperature is a fundamental environmental factor, and knowledge about its effects on cells is important as it affects various processes inside the cell, and also cells have temperature‐specific channels. TRPM8 is a potent regulator of osteogenic differentiation of MSCs (Acharya, Kumar, et al., 2021; Henao et al., 2021) and also affects the differentiation of T cells, monocytes, neurons, and keratinocytes (Acharya, Tiwari, et al., 2021; Bidaux et al., 2015; Hornsby et al., 2022; Oz & Celik, 2022). Therefore, its effect on multipotency and differentiation potential of MSCs is interesting. In this study, we examined the effects of TRPM8 on myogenic differentiation of MSCs, which is different from osteogenic differentiation, a pathway, that has already been reported to be affected by TRPM8. Furthermore, we aimed to clarify the effects of low temperature as well as agonists as a stimulus. This is the first study to examine the effect of low temperature (cooling) and agonist stimulation on myogenic differentiation of MSCs.

## 2 MATERIALS AND METHODS

### 2.1 In vitro MSCs culture

C57BL/6 mouse bone marrow‐derived MSCs (MUBMX‐01001: KSC Co., Ltd, Kyoto, Japan) were cultured in an RPMI‐1640 medium (ThermoFisher Scientific, Tokyo, Japan) with 10% fetal bovine serum (FBS: Nichirei, Tokyo, Japan) at 37°C in 5% CO_2_.

### 2.2 Myogenic differentiation of MSCs and interventions

Three times passaged MSCs were seeded into 35‐mm dishes at 5000 cells/dish with reference to the previous study (Wakitani et al., 1995). Twenty‐four hours postseeding, the cells were cultured for 24 h in an RPMI‐1640 medium containing 10% FBS and 10 μM 5‐azacytidine (Sigma‐Aldrich, St. Louis, MO). After the differentiation induction treatment, the medium was changed to RPMI‐1640 with 10% FBS and placed in a CO₂ incubator for 6 h before immunohistochemistry was performed.

To determine the effect of TRPM8 activation on myogenic differentiation, the cells were cultured in differentiation induction media containing 25 μM menthol (63,660: Sigma‐Aldrich, St. Louis, MO), a TRPM8 agonist (McKemy et al., 2002; Peier et al., 2002).

To determine the effect of cooling stimuli on myogenic differentiation, a cooling plate (HMC‐12W‐0100: Hayashi‐Repic, Tokyo, Japan) was set outside a CO₂ incubator, and the dishes were kept on the plate 5 min after differentiation induction. The cooling plate temperature was set at 17°C, based on the temperature range at which cooling‐induced [Ca^2+^]i increase via TRPM8 activation was observed in the current study (Figure 2). To ensure uniform conditions for each experiment, all groups spent the longest cooling time in the cooling experiment in media containing 20 mM HEPES (Santa Cruz Biotechnology, Dallas, TX) in an uncontrolled CO₂ environment.

To determine the involvement of TRPM8 in promoting myogenic differentiation by 60 min of cooling stimulus, the cells were cultured in media containing 40 μM AMTB (SML0103: Sigma‐Aldrich, 20 mM in dimethyl sulfoxide), a TRPM8 antagonist (Lashinger et al., 2008).

### 2.3 Evaluation of myogenic differentiation

To assess skeletal myogenic differentiation of MSCs, immunohistochemistry was performed using MyoD, a specific marker for skeletal myogenic differentiation, as previously described (Geng et al., 2009; Jia et al., 2018). Cells were fixed with 4% paraformaldehyde for 10 min and permeated with 0.2% Triton X‐100 for 5 min at room temperature. After washing with phosphate‐buffered saline (PBS), cells were blocked with 3% goat serum in PBS for 30 min at room temperature. Cells were incubated with 1:50 diluted anti‐MyoD antibody (18943‐1‐AP: Proteintech, Rosemont, IL) overnight at 4°C. Cells were washed with PBS and then incubated with 1:200 diluted anti‐rabbit IgG Alexa Fluor 647 (Thermo Fisher Scientific) in the dark for 1 h at room temperature. Cells were washed with PBS and mounted with VECTASHIELD Mounting Medium with DAPI (Vector Laboratories, Newark, CA). Cells were observed and photographed with a fluorescence microscope (BZ‐9000: Keyence, Osaka, Japan). Images were taken from two randomly selected views for each treatment in each experiment. All photos from the same experiment were processed in an identical manner, including brightness adjustment and contrast enhancement using an analytical application (BZ‐II Analyzer: Keyence). The percentage of MyoD (red)‐positive cells per DAPI (blue)‐positive cells was calculated based on the average of the two different fields. In this analysis, the order in which images were analyzed was not random, but one person was in charge so that no one could tell which group the image was from.

### 2.4 Calcium imaging

To observe variations in intracellular Ca^2+^ concentration ([Ca^2+^]i), MSCs were incubated in media containing calcium indicator fura‐2 (5 μM fura‐2am, 0.04% Pluronic F‐127, 1 mM Probenecid in Hanks' Buffer with 20 mM HEPES [HHBS]) for 60 min in a CO_2_ incubator. The medium was then replaced with HHBS, and the cells were observed under a microscope on a cooling plate (HMC‐12W‐0100: Hayashi‐Repic) set at 37°C. For fluorescence photography, the excitation durations in no‐delay mode for 340 and 380 nm were set as 500 and 300 ms, respectively. The ratio (340 nm/380 nm) was calculated by using image‐capture software (NIS‐Elements Advanced Research: Nikon, Tokyo, Japan) from 510 nm fluorescence photographs taken by a CMOS camera (ORCA‐Flash4.0; Hamamatsu Photonics, Shizuoka, Japan) and shown as a rainbow gradation without need for any additional processing.

To determine the involvement of TRPM8 and extracellular Ca^2+^, we set up three groups: a control group cultured in HHBS‐containing vehicles, a group cultured in HHBS containing 40 μM AMTB, a TRPM8 antagonist, and a group cultured in HHBS from which CaCl₂ was excluded. The temperature of the cooling plate was lowered from 37°C to 2°C after 5 min of replacement with each culture media, and the plate temperature was returned to 37°C after the temperature of media fell below 10°C. A temperature sensor (IT‐18: Physitemp, Clifton, NJ) was placed in the medium. Photographs were taken once every 5 s, and the ratio was calculated from the entire field of view (approx. 830 μm^2^).

### 2.5 Statistics

The difference between menthol‐treated and nontreated groups was determined using paired t‐test. The difference between the two factors of temperature and media was examined by a two‐way analysis of variance (ANOVA) followed by Dunnett's test. The difference between cooling times was examined by a one‐way ANOVA followed by Dunnett's test. The difference between the two factors of cooling and antagonist was examined by a two‐way ANOVA followed by Tukey's multiple comparison test. Statistical significance was set at *p* < 0.05.

## 3 RESULTS

### 3.1 Effect of menthol, a TRPM8 agonist on skeletal myogenic differentiation of MSCs

We added menthol in 5‐azacytidine contained media and then cultured MSCs in it to determine the effect of TRPM8 agonist on skeletal myogenic differentiation of MSCs.

After 24 h of 5‐azacytidine loading, the media was replaced with an RPMI‐1640 medium containing 10% FBS, and 6 h later, the percentage of MyoD‐positive cell nuclei was calculated.

**FIGURE 1:**
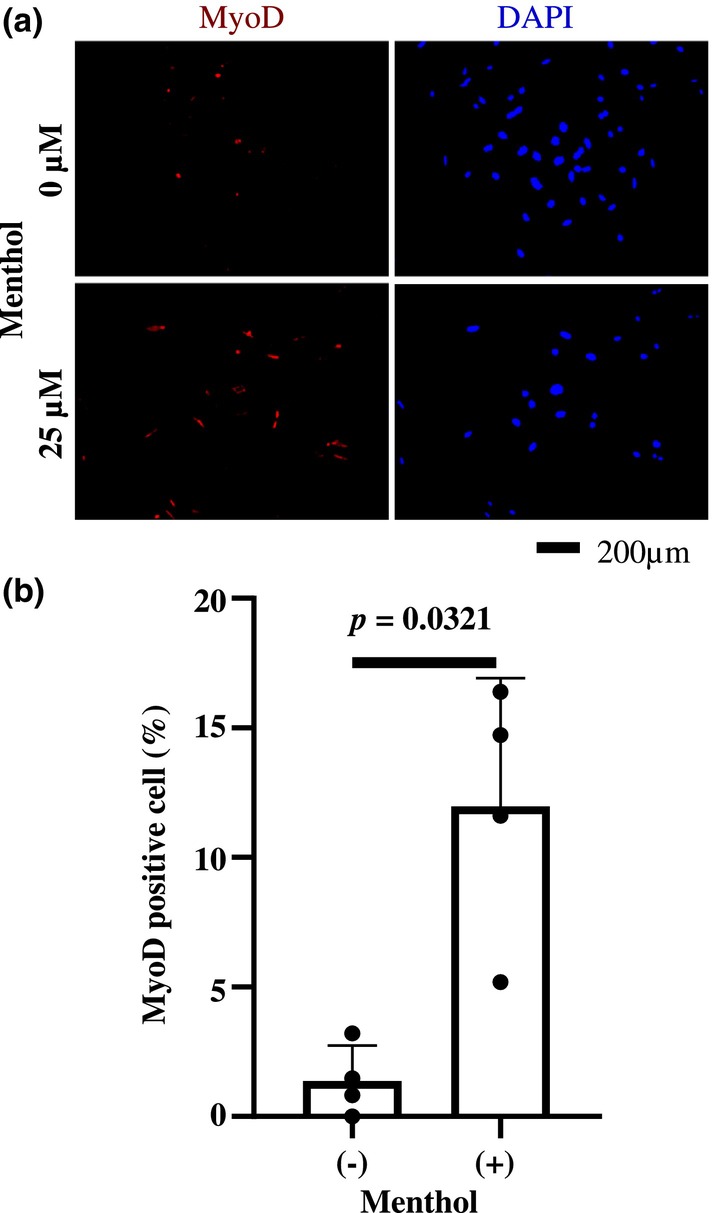
Menthol promotes differentiation of MSCs into myogenic cells. The pictures (a) show MyoD expression and DAPI‐stained images of MSCs from the same field of view in the absence and presence of menthol added in the myogenic differentiation media. The graph (b) shows the percentage of MyoD‐positive (red) cells per DAPI‐positive (blue) nuclei and was obtained from four independent experiments. The results are shown as mean ± standard deviations. Significant difference is set at *p* < 0.05.

Figure 1b shows that menthol‐treated group had significantly higher percentage of MyoD‐positive cell nuclei (12.0 ± 2.14%) than nontreated group (1.38 ± 0.59%).

### 3.2 Cooling‐induced TRPM8 activation increases [Ca^2+^]i in MSCs

We treated cells with three different conditions to determine whether TRPM8 and intracellular Ca^2+^ are affected by cooling stimulus: a control group with vehicle medium, a group treated with AMTB, and a group cultured in the absence of extracellular Ca^2+^.

**FIGURE 2:**
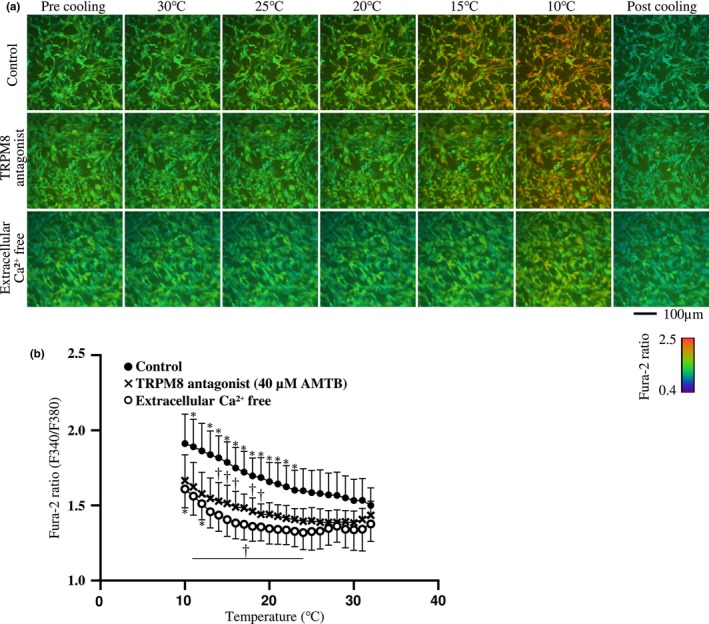
Involvement of TRPM8 and extracellular Ca^2+^ in cooling‐induced elevation of [Ca^2+^]i in MSCs. The pictures (a) are representative examples of changes in [Ca^2+^]i (rainbow gradation) when MSCs were loaded with fura‐2, a calcium indicator, and the temperature was lowered and raised, showing the center of the camera's field of view (upper row: control, middle row: loaded with TRPM8 antagonist (40 μM AMTB), lower row: extracellular Ca^2+^‐free condition). The graph (b) shows the variation of the average fura‐2 ratio over the entire field of view with change in temperature. The mean values ± standard deviations are shown for each of the four sessions of the different dishes. Statistical significance was at *p* < 0.05. Comparisons were made with the Control group or 32°C values. * vs. 32°C in same group and † vs. Control in same temperature.

Variation in [Ca^2+^]i was determined by the changes in Fura‐2 fluorescence ratio. Figure 2b shows that the control group had a significant increase in [Ca^2+^]i when the medium was cooled to 11°C and 13–23°C compared with 32°C (i.e., before cooling). The TRPM8 antagonist group showed significantly lower [Ca^2+^]i when cooled to 14–16°C and 18–19°C compared with the control group. In the extracellular Ca^2+^‐free group, a significant increase in [Ca^2+^]i was observed after cooling to 10°C and 12°C compared with before cooling, but [Ca^2+^]i was lower at 11–24°C compared with the control group.

### 3.3 Cooling promotes skeletal myogenic differentiation of MSCs

To examine the effect of cooling stimuli on myogenic differentiation of MSCs, the cells were cooled outside the CO₂ incubator after replacement with differentiation‐inducing medium containing HEPES. There were no significant differences in cell counts by DAPI between groups (*p* = 0.3891, data not shown).

**FIGURE 3:**
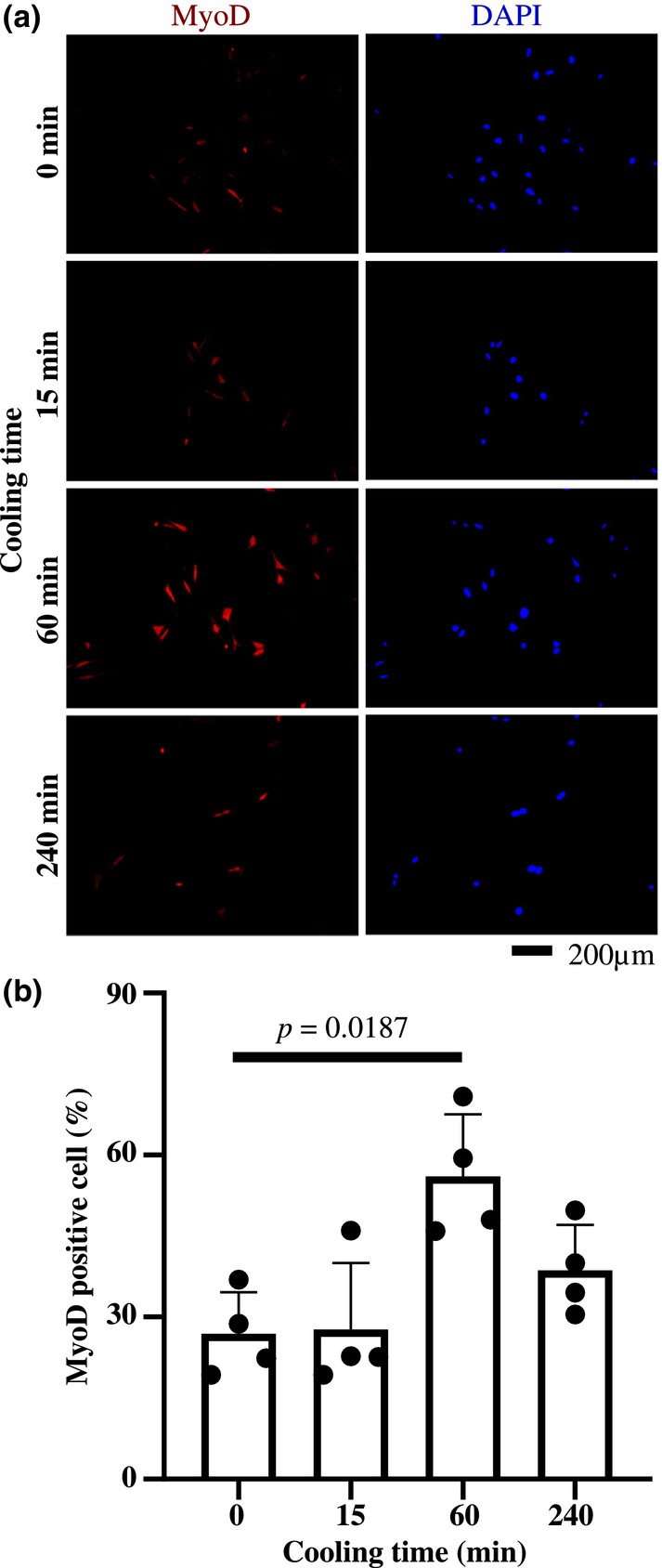
Effect of cooling on myogenic differentiation of MSCs. The pictures (a) show MyoD expression and DAPI‐stained images of MSCs in the same field of view cultured in myogenic differentiation induction media exposed to different cooling. The graph (b) compares the percentage of MyoD‐positive (red) cells per DAPI‐positive (blue) nuclei and was obtained from four independent experiments. The results are shown as mean ± standard deviations. Statistical significance was at *p* < 0.05. Comparisons were made with 0 min.

Figure 3b shows that the 60‐min cooling group (56.06 ± 4.98%) had a significantly higher percentage of myogenic differentiated cells than the no (0 min) cooling group (26.86 ± 3.37%).

### 3.4 Cooling‐promoted MSCs differentiation involves TRPM8 activation

**FIGURE 4:**
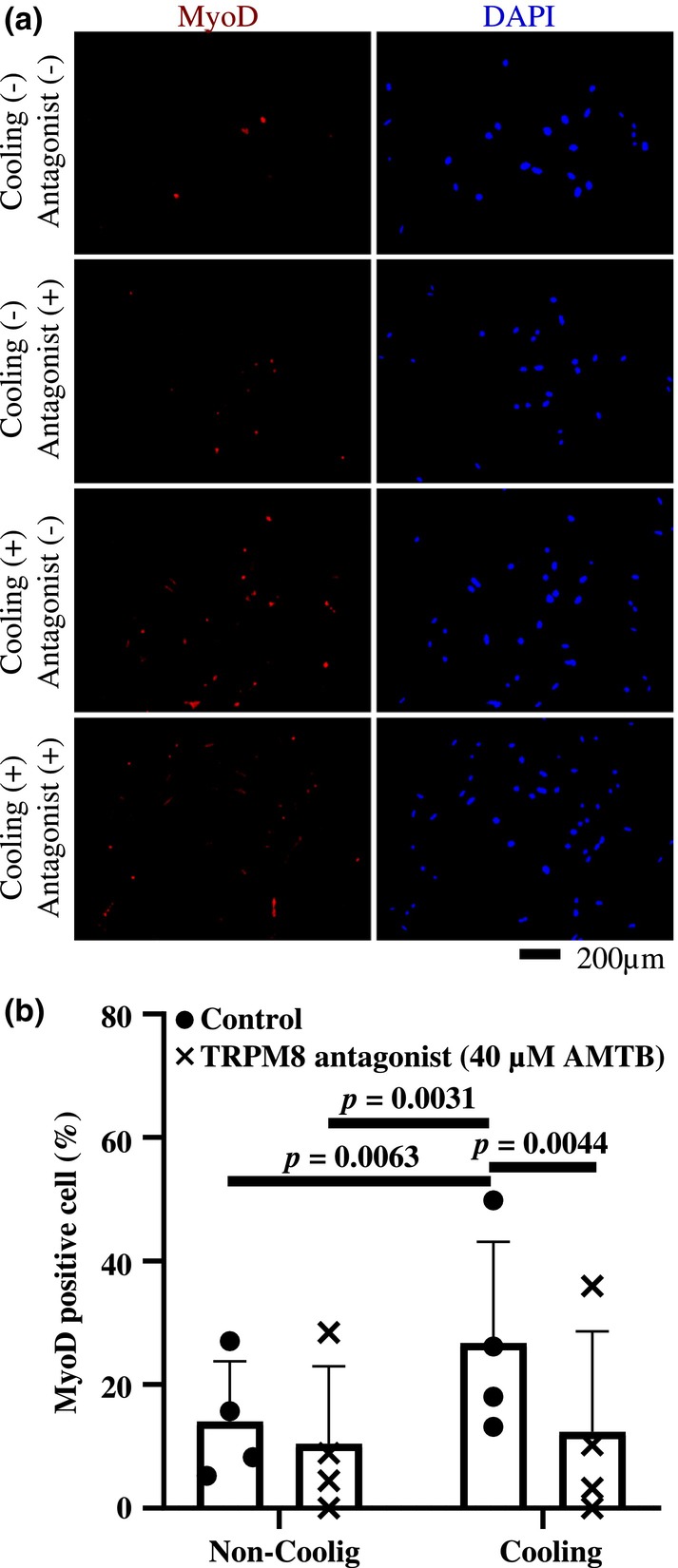
Effect of TRPM8 antagonist on myogenic differentiation of MSCs with and without cooling. The pictures (a) show MyoD expression and DAPI‐stained images of MSCs in the same field of view with and without cooling cultured in myogenic differentiation induction media containing TRPM8 antagonist (40 μM AMTB). The graph (b) compares the percentage of MyoD‐positive (red) cells per DAPI‐positive nuclei (blue) and was obtained from four independent experiments. Shown are means and standard deviations. Statistical significance was at *p* < 0.05.

We wanted to determine if cooling‐promoted skeletal myogenic differentiation of MSCs involves TRPM8. Therefore, we performed the experiment as shown in Figure 4 in the presence of a TRPM8 antagonist, AMTB.

Figure 4b shows that cooling‐promoted myogenic differentiation (*p* = 0.0063) is suppressed by TRPM8 antagonist (*p* = 0.0044). This proves that activation of TRPM8 is involved in cooling promoted MSCs differentiation.

## 4 DISCUSSION

In this study, we found that cooling‐induced TRPM8 activation promotes 5‐azacitizine‐induced myogenic differentiation of murine bone marrow‐derived MSCs. This is the first study that demonstrates that TRPM8 activation is involved in differentiation pathways other than osteogenic differentiation as previously reported (Acharya, Kumar, et al., 2021; Henao et al., 2021). The advantage of the current study is that single‐cell type in vitro experiments can exclude indirect effects of other factors, such as hormones and other secretory factors, that might occur in vivo. Thus, we can conclude that the pro‐differentiation adaptation is caused solely by the MSCs' own response to the cooling stimulus.

### 4.1 Menthol, a TRPM8 agonist, promotes myogenic differentiation of MSCs

Brief exposure to 5‐azacitizine induces myogenic differentiation of MSCs (Wakitani et al., 1995; Zhang et al., 2019). The rate of myogenic differentiation induced by 5‐azacytidine, as measured by the percentage of MyoD‐expressing cells, is low (Geng et al., 2009; Jia et al., 2018).

Menthol is a typical pharmacological agent that activates TRPM8 (McKemy et al., 2002; Peier et al., 2002). The osteogenic differentiation of MSCs is enhanced by the addition of menthol to the differentiation induction media (Henao et al., 2021). In this study, we observed that percentage of MyoD‐positive cells increased when menthol was added to myogenic differentiation induction media. These results suggest that myogenic differentiation of MSCs is promoted through TRPM8 activation and that TRPM8 is involved in multiple differentiation pathways of MSCs.

### 4.2 Cooling promotes MSCs myogenic differentiation via TRPM8 activation

The purpose of this study was to verify whether cooling stimulation can promote myogenic differentiation through TRPM8 activation. TRPM8 is a temperature‐sensitive channel that is activated by cold exposure and increase [Ca^2+^]i (McKemy et al., 2002; Peier et al., 2002). A rise in [Ca^2+^]i is thought to activate calcium‐activated potassium channels that regulate MSCs differentiation (Pchelintseva & Djamgoz, 2018).

Ca^2+^ imaging of MSCs in this study showed that [Ca^2+^]i is increased below 23°C. Peier et al. (2002) reported 23°C as the upper threshold at physiologically relevant temperature in TRPM8‐expressing cells and [Ca^2+^]i elevation in cooling to 15°C is abolished by removal of extracellular Ca^2+^. In the present study, extracellular Ca^2+^ free condition suppressed the [Ca^2+^]i increase. On the other hand, at lower temperatures (10°C and 12°C), even in the absence of extracellular Ca^2+^, an increase in [Ca^2+^]i was observed. TRPM8 is expressed on the endoplasmic reticulum in addition to the plasma membrane in human bone marrow MSCs (Henao et al., 2021). McKemy et al. (2002) showed that menthol, a TRPM8 agonist, acts in the absence of extracellular Ca^2+^. To investigate the involvement of TRPM8 activation in the [Ca^2+^]i increase, changes in [Ca^2+^]i were observed in the presence of AMTB, a TRPM8 antagonist. We observed that the increase in [Ca^2+^]i was suppressed and undetectable. These results suggest that increase in [Ca^2+^]i in MSCs during cooling is due to TRPM8 activation.

The effect of cooling time on the myogenic differentiation of MSCs was examined at cooling temperatures that activate TRPM8 (i.e., 17°C). Van't Hoff's law states that a decrease in temperature decreases the rate of enzymatic reaction. Therefore, in the present study, we applied cooling only during the early phase of differentiation induction, and it was found that cooling for 60 min promoted myogenic differentiation of MSCs. Furthermore, we showed that cooling‐induced promotion of myogenic differentiation is not seen in the presence of AMTB, a TRPM8 antagonist. These results indicate that cooling is an effective means of activating TRPM8, which then regulates MSC differentiation.

Thermal stimulation by cooling is an approach that can be applied to MSCs transplanted in vivo in regenerative medicine since MSCs have the ability to differentiate into the target cell lineage by phagocytosis (Wakao et al., 2022). However, there is a limitation in applying this method in vivo, that is, the difference between the temperature threshold at which TRPM8 is activated in vitro and in vivo. The temperature threshold is affected by various factors such as pH and phosphatidylinositol 4,5‐bisphosphate (PIP_2_) (Andersson et al., 2004; Rohacs et al., 2005). For example, in a study using human embryonic kidney 293T cells, the upper‐temperature threshold was 28°C when the ambient temperature was 30°C, whereas it was 35°C when the ambient temperature was 40°C, suggesting that PIP_2_ is involved (Fujita et al., 2013). In addition, the cooling intervention in this study was performed outside the incubator where the CO₂ concentration, which affects pH, was not controlled. More stringent control of these cellular environmental factors is needed to establish a cooling approach that induces activation of TRPM8, which promotes MSC differentiation in an in vivo environment.

In summary, this study demonstrated that thermal stimulation at 17°C for 60‐min promotes myogenic differentiation of MSCs via TRPM8 activation. Thermal stimulation is a promising approach that can be used to demonstrate the differentiation diversity of MSCs in regenerative medicine.

## AUTHOR CONTRIBUTIONS

Conception and design of the work: Ryo Takagi, Junya Takegaki, Shion Osana, Yutaka Kano, Satoshi Konishi, and Satoshi Fujita. Acquisition and analysis: Ryo Takagi, Junya Takegaki, Shion Osana, and Yutaka Kano. Interpretation of data: Ryo Takagi, Junya Takegaki, Shion Osana, Yutaka Kano, Satoshi Konishi and Satoshi Fujita. Writing the original draft: Ryo Takagi. Review and editing: Junya Takegaki, Shion Osana, Yutaka Kano, Satoshi Konishi, and Satoshi Fujita. All authors approved the final version of the manuscript.

## FUNDING INFORMATION

This work was supported by a Grant‐in‐Aid for Early‐Career Scientists (Ryo Takagi, no. 21K17452) from the Japan Society for the Promotion of Science and the Ritsumeikan Global Innovation Research Organization project at Ritsumeikan University.

## CONFLICT OF INTEREST STATEMENT

No conflicts of interest, financial, or otherwise, are declared by the authors.

## ETHICS STATEMENT

The animal‐derived cells used were purchased and did not require approval by an ethics committee to perform the experiments.

## Data Availability

The data that support the findings of this study are available from the corresponding author upon reasonable request.
